# The Impact of Heat Waves on Occurrence and Severity of Construction Accidents

**DOI:** 10.3390/ijerph14010070

**Published:** 2017-01-11

**Authors:** Rameez Rameezdeen, Abbas Elmualim

**Affiliations:** School of Natural and Built Environments, University of South Australia, Adelaide 5001, Australia; Rameez.Rameezdeen@unisa.edu.au

**Keywords:** construction, heat wave, accident, severity, compensation claim, Adelaide

## Abstract

The impact of heat stress on human health has been extensively studied. Similarly, researchers have investigated the impact of heat stress on workers’ health and safety. However, very little work has been done on the impact of heat stress on occupational accidents and their severity, particularly in South Australian construction. Construction workers are at high risk of injury due to heat stress as they often work outdoors, undertake hard manual work, and are often project based and sub-contracted. Little is known on how heat waves could impact on construction accidents and their severity. In order to provide more evidence for the currently limited number of empirical investigations on the impact of heat stress on accidents, this study analysed 29,438 compensation claims reported during 2002–2013 within the construction industry of South Australia. Claims reported during 29 heat waves in Adelaide were compared with control periods to elicit differences in the number of accidents reported and their severity. The results revealed that worker characteristics, type of work, work environment, and agency of accident mainly govern the severity. It is recommended that the implementation of adequate preventative measures in small-sized companies and civil engineering sites, targeting mainly old age workers could be a priority for Work, Health and Safety (WHS) policies.

## 1. Introduction

It has been widely documented that exposure of construction workers to excessive heat stress has a paramount impact on their health and well-being as well as their productivity [1,2]. This exposure to heat stress has further been exacerbated by the increased frequency of heat waves due to global climate change [3,4]. The exposure of construction workers to heat stress is seen as a growing challenge to the construction industry [5]. Despite the implementation of various strategies and policies within the sector, an increase in heat stress related morbidity and mortality is widely reported [6]. Heat stress is the largest cause of weather-related deaths in the United States [7]. Analysis of mortality due to heat stress during 2000–2010 revealed construction workers are extremely vulnerable to heat stress with the second highest risk rate among all industries in the United States [7]. Workers belonging to small companies (with fewer than 10 employees) had the highest fatality rate, prompting the authors to suggest targeted heat stress prevention interventions in small firms. Small companies are less likely to implement heat stress prevention procedures or monitoring guidelines due to capacity constraints compared to their larger counterparts [8]. 

The assessment of the impact of heat waves on construction workers is a growing challenge for the South Australian Construction industry, with adverse impacts on project delivery. It is projected that by 2070 heat waves will triple in South Australia [1]. Construction workers, working on office environments or sites, will face various challenges in finding the right balance between their required duties and personal safety [2]. The influence of heat stress on unintentional accidents and injuries is an increasingly important research question in the face of global warming and climate change [9]. The combination of heat and humidity can put workers’ health at risk, especially during prolonged period of exposure. Evaporative sweat (heat loss) is seen as the mechanism to reduce heat stress and other risks of injury [4]. Evaporative heat loss through sweat can be restricted by age, gender, low air movement, and protective equipment [2,4]. In such cases, a positive body heat and related skin temperatures will increase, leading to heat illness and death in some circumstances. Due to the physical nature of construction work, especially outdoors and on sites, construction workers face a greater risk due to high metabolic rate causing increases in body temperature [10]. The combined metabolic and environmental heat loads challenge the bodies’ cooling mechanisms in these workers [7]. For example, roofers perceived an extra 10 °C temperature while working on roofs directly exposed to sunshine [11]. With the threat of heat wave events, the situation is much worse, even if the level of activity is low; it represents a high risk of heat stress, ill-health, and mortality. It was established that a core body temperature above 40 °C will lead to heat exhaustion. In return, heat exhaustion poses a great threat to life and can lead to complete failure of central nervous thermoregulatory system [12]. Simmons et al. (2008) argued that minimum increases in body temperature can be detrimental to workers’ performance, cognitively as well as physically [12]. Increased sweating and high level of heat sensation can cause discomfort and distress, causing distraction and other behavioural changes which can result in accidents and injuries. High levels of sweat during heat waves may result in dehydration. Lieberman (2007) suggested that a 2% or more loss of body weight due to dehydration will result in significant reductions in visual-motor tracking, loss of short term memory, and attention [13]. It is evident that heat stress can have progressive effects on workers’ health ranging from heat rash, heat cramp, heat fainting, heat exhaustion to heatstroke, leading to morbidity and mortality [2].

Causes of construction accidents have been extensively studied with a number of theories explaining the root causes [14]. Uncovering root causes of accidents is very important for the construction industry as it is mainly project-based and transitionary in character, where Work, Health and Safety (WHS) measures are very hard to implement [15]. Site conditions, type of work, and work environment are considered to be influential in accident causation [16,17,18]. Heat stress can induce other construction accidents through physical fatigue, impaired mental capacity, and misuse of inconvenient personal protective equipment (PPE) [19]. Using a systems approach, Rowlinson and Jia (2015) studied the causation of heat illnesses in Hong Kong [20]. Heat illness is considered to be a special case where the victim is often the agent of its cause and prevention largely rests with the victim as well as the institutions surrounding the construction process [20]. The masculine culture of the construction industry was found to be the major cause of heat stress illnesses, where workers often tend to underestimate the risk while overestimating their capacity to cope with it [21]. In addition to factors causing construction accidents, factors that influence its severity are also important in order to plan preventive measures. Using worker characteristics, type of work, and the work environment, Dumrak et al. (2013) found that age, experience, gender, and language used by the worker; organization size, project size, and project location; mechanism of accident; and body location of the injury are factors that influence the severity of an accident [22]. 

Due to global warming, a large number of countries are at risk of experiencing intense and frequent heat waves. These countries have to assess the human health vulnerability to climate change in order to plan and implement mitigation measures to avoid large calamities. Therefore, more empirical evidence is needed to understand the impact of heat waves on occupational accidents, especially for industries such as construction. This paper aims to investigate the impacts of heat waves on construction accidents using a large number of compensation claims compiled by SafeWork South Australia. These data provide an insight into many variables related with an accident as reported by the victim. The study focuses mainly on the occurrence and severity of accidents during the 2002–2013 period in South Australia. In practical terms, uncovering potential impacts of heat waves on accidents and the groups and activities that are vulnerable could help improve the effectiveness of WHS measures implemented on sites. It will also enhance the construction organizations’ understanding of the potential impacts, thereby allowing them to be able to implement policies on heat stress management more stringently. Additionally, an increased understanding would enable organizations to channel their energies across the groups and activities having the highest vulnerability. As a result, the implementation of WHS measures would be more likely to be successful.

## 2. Research Method

### 2.1. Injury Data

The data for this study was obtained from SafeWork South Australia (SafeWork SA), which is the state government’s occupational health, safety, and welfare agency. It collates workers’ compensation claims data obtained from WorkCover SA into a database for policy analysis. WorkCover SA is a government agency that is responsible for the prevention and compensation of occupational accidents and diseases in South Australia. It is entrusted with the administration and regulation of the Workers Rehabilitation and Compensation Act 1986 and the South Australian Workers Rehabilitation and Compensation scheme. Australia introduced the Type of Occurrence Classification System (TOOCS2) as a coding guideline for the recording of compensation claims [1]. The database is a large convenience sample representative of the South Australia’s working population. This database is made available to researchers after signing an agreement with regard to its use specifically to safeguard the confidentiality of victims. The data contained 29,438 workers’ compensation claims reported during 1 July 2002–31 June 2013 in South Australia for the industry category “construction” based on the Australian and New Zealand Standard industrial Classification (ANZSIC) coding. The study is restricted to injuries occurred within the Adelaide Metropolitan Region, and those injuries were extracted using the location postcode.

### 2.2. Injury Severity

The classification of the severity of an accident is determined by the severity of injuries suffered by the victim(s). Different methods and classifications of injury severity were used in past research. National Patient Safety Agency (NPSA) of the United States categorizes injuries into five groups based on the treatment necessitated for the victim ([23], p. 6). A “negligible” injury is one which the victim does not need treatment, whereas a “minor” injury will only necessitate minor treatment. A “moderate injury” requires the victim to undergo professional treatment and care. A “major injury” is one which the victim suffered a long-term incapacitation. The highest in the scale, “catastrophic” refers to injuries leading to death, multiple permanent incapacities, or irreversible health effects. Using a similar methodology, Aneziris et al. (2012), classified injuries into three groups: recoverable, non-lethal permanent, and lethal [24]. Between NPSA and Aneziris et al. the former is more practical as it has many intermediate levels of non-fatal injuries. Nevertheless, both classifications suffer from potential data collection issues as the treatment records would only be available through hospital or health sources. Therefore, accidents which did not use treatment outcomes will not be captured through these classifications. In such context, proxy measures of injury severity would become very useful. Dumrak et al. (2013) used a combination of hospitalization and number of lost days from work as a means of classifying accident severity into six groups [22]. Using a similar philosophy, the present study employ two measures for accident severity: injury classified as “minor” and “major” based on number of lost days from work, and the cost of compensation (AUS$). 

A “minor” injury is defined as one with zero lost days while a “major” injury necessitates the victim to be absent from work for recovery. As the cost of recovery is directly proportional to the amount claimed as compensation, the dollar value is used as an alternative measure of accident severity. These two proxy severity measures are useful in three ways. First, they do not depend on treatment to physical injuries and need not be from hospital or health sources. Therefore, it eliminates all potential biases of non-capture of minor injuries that never sought treatment or hospitalization. Second, it captures non-physical injuries, such as socio-psychological, which are very conveniently being neglected in construction WHS. However, due to increasing numbers of such injuries in workplaces, researchers are now paying very close attention to socio-psychological health at workplaces. As they cannot be ignored anymore, proxy severity classifications become very useful in capturing those injuries; Third, proxy measures are not biased towards either end of the severity continuum, as most of the treatment-based methods tend to be, and provide a balanced representation of accident severity.

### 2.3. Heat Wave and Meteorological Data

Lundberg et al. (2013, p. 4) defined heat waves as “extended periods of unusually high atmospheric related heat stress, which cause temporary modification in lifestyle and which may have adverse health consequences for the population” [25]. There are a number of interpretations of the threshold limit beyond which a period is considered as a heat wave period. The Australian Bureau of Meteorology (BoM) criterion is either five or more consecutive days of maximum temperature in excess of 35 °C or three or more consecutive days of temperature in excess of 40 °C. Other definitions include: greater than two consecutive days of maximum temperature in excess of 37 °C [26]; greater than three consecutive days of maximum temperature in excess of 35 °C [1]. Daily maximum and minimum temperatures for the study period were obtained from Kent Town weather station (located 34.93° S, 138.53° E; station number 23090) of the Australian Bureau of Meteorology as a representation of the Adelaide metropolitan region. These temperatures were verified with five more stations around the city representing northern, western, southern, north eastern, and south western suburbs of the city. Due to the complications involved in using two definitions suggested by the Australian Bureau of Meteorology, this study used the definition of Xiang et al. [1]. Accordingly, 29 heat waves occurred during the study period ranging from 3 to 15 days with a mean value of 5.1 days per heat wave. Compensation claims recorded during these heat wave periods were extracted from the SafeWork SA database for analysis.

### 2.4. Study Design

This study uses a quasi-experimental study design to empirically test the impact of heat waves on occurrence and severity of construction accidents. Compensation claims recorded during the heat wave periods were compared with those during similar “control periods”. The control periods were selected based on the methodology used in Case Crossover analysis (see [27] for further details). The control period comprised of the same week days and the duration of the heat wave either prior to or after the event with a gap of seven days between the two. The control period was the first available prior to the event which ensured most of the climatic and other confounding factors are controlled. However, if another heat wave prevented selecting the immediate pre-event period, either a post-event or a period within the half of the month was used. Worker characteristics, type of work, and work environment were used as variables that affect the occurrence and severity of an accident based on [22]. Heat waves increase the ambient heat level of the work environment, especially for work activities that are outdoors. Even the indoor work activities could be affected by increased ambient heat. 

## 3. Results

### 3.1. Characteristics of Worker

The relationship between worker characteristics; represented by age, experience, and gender; and occurrence of accidents during heat wave and control periods are shown in Table 1. It shows a slightly higher representation of age groups younger than 35 and older than 55 among accidents during heat wave periods. Experienced workers and male workers were also slightly over-represented among accidents during the heat wave periods. Nevertheless, all three factors have been found not to have a statistically significant association with number of accidents (with χ^2^ = 8.974, d.f. = 5, *p* = 0.110 for age; χ^2^ = 0.522, d.f. = 1, *p* = 0.470 for experience; and χ^2^ = 2.850, d.f. = 1, *p* = 0.091 for gender). The Chi Square statistic (χ^2^) compares the tallies or counts of categorical variables between two (or more) independent groups to determine whether there is a significant association among these variables. The degrees of freedom (d.f.) is defined as (r − 1) × (c − 1) where r is the number of levels for one categorical variable and c is the number of levels for the other categorical variable. The *p*-value (*p*) is the probability of observing a sample statistic as extreme as the test statistic.

With regard to severity of accidents, Table 2 shows their link to heat waves. Workers above 55 years of age were over-represented among major accidents during heat wave periods compared to control periods (15.2% for former against 11.2% for latter). The average expenditure for accidents among this cohort of workers was AUS$26,381 during heat waves compared to AUS$12,747 during control periods. This shows the disproportionate effect of heat waves among older workers. Similarly, new workers suffered a relatively higher proportion of major injuries during heat wave periods (14.9% during heat waves against 12.1% during control periods). New workers in this study are defined as those who have less than one year of experience working in construction projects. The mean expenditure for accidents among new workers was AUS$23,806 during heat waves compared to AUS$16,733 during control periods. Both indicators point to the vulnerability of new workers during heat waves. With regard to gender, female representation in the South Australian construction workforce is about 13% according to the 2011 census [28]. Table 2 shows that male workers have suffered a disproportionately higher proportion of major accidents compared to female workers during heat waves. Male workers’ average expenditure was slightly higher during the heat wave periods than the control periods. The above result shows the groups of workers that are relatively more vulnerable to severe injuries during heat waves. 

The relationship between age of worker and injury severity has been studied by many past researchers. They all agree that older age workers are highly vulnerable to severe construction accidents [29,30]. While confirming this general view, this research in addition shows that this cohort is more vulnerable to severe accidents during heat waves. A number of researchers have highlighted that older people are more likely to develop severe health problems during heat waves than the younger population. Past research has shown new workers, who are less than one month working in a site, are relatively more vulnerable to severe injuries on construction sites [16,31]. New workers tend to be more vulnerable to heat related injuries as they might not be acclimatized to the heat and the exertion required for the job [7,32].

### 3.2. Type of Work

The type of work undertaken by the worker was investigated with two variables, namely, sub-sector and occupation. The Australian census classifies construction workers into four sub-sectors: general construction, building, civil engineering, and construction services. According to the 2011 census, 2.3%, 30.5%, 7.5%, and 59.65% of construction workers in South Australia were belonging to the above four sub-sectors, respectively [28]. It shows that construction services and building sub-sectors combined employ the majority of the construction workforce in South Australia. A number of trades and occupations are involved in the delivery of a construction product. These occupations are categorized according to the Type of Occurrence Classification System (TOOCS2). The relationship between occurrence of accidents among these categories of workers and heat waves is shown in Table 3. It shows a very high proportion of workers in the civil sub-sector to be represented in accidents during heat waves. Compared to the control period, the percentage is more than double during the heat wave period (6.4% against 15.6%). With regard to occupation, there is no significant difference between the two periods, with a slight over-representation of bricklayer, carpenter, electrician, mechanic, and plant operator among accidents during heat waves. Out of these two factors, only the sub-sector is found to have a statistically significant association with number of accidents (with χ^2^ = 31.660, d.f. = 3, *p* = 0.000 for sub-sector; and χ^2^ = 7.786, d.f. = 14, *p* = 0.932 for occupation). Therefore, the impact of heat waves on the civil engineering sub-sector becomes a very serious concern for construction companies and WHS authorities.

The relationship between type of work and severity of injuries during heat wave and control periods are shown in Table 4. Workers belonging to civil engineering sub-sector seem to have suffered disproportionately during heat waves due to severe injuries. However, the mean cost of injuries of this sub-sector during heat wave periods is far below that of control periods. In terms of expenditure per worker-injury, general construction and building sub-sectors show higher values during heat waves.

### 3.3. Work Environment

The work environment is one of the major determinants of the severity of an accident. Accordingly, size of company, location of worksite, and agency of accident were included as representatives of the work environment for this study. The relationship of these factors with occurrence of accidents is summarized in Table 5. Workers belonging to small and medium sized companies were slightly over-represented among accidents during the heat wave periods. There is no major difference between projects located in the Central Business District (CBD) and suburbs with regard to occurrence. The share of accidents for agencies such as equipment, environment, vehicle, and work platform were higher during the heat waves compared to the control period. Nevertheless, all three factors have been found not to have a statistically significant association with number of accidents (with χ^2^ = 1.840, d.f. = 2, *p* = 0.398 for size of company; χ^2^ = 0.677, d.f. = 1, *p* = 0.703 worksite location; and χ^2^ = 10.403, d.f. = 8, *p* = 0.238 for agency of accident).

In terms of severity of injuries, as shown in Table 6, small companies had a proportionately higher share of severe injuries based on both indicators. The difference between locations of worksite (Adelaide CBD vs. suburbs) did not show a major difference between heat wave and control periods. However, mean expenditure of CBD based worksites had a relatively higher value during heat wave periods. Agencies such as equipment, environment, vehicle, and work platform were over-represented among major accidents while the mean cost of injury was higher for agencies such as structure, electricity, environment, small tool, and vehicle.

## 4. Discussion

This study looked at the differences of injuries suffered by construction workers during heat wave and control periods to identify groups and activities that are vulnerable to heat waves. It highlights some important implications for the construction community and WHS authorities. The number of accidents reported during heat wave periods is less compared to control periods, suggesting some control measures are in operation in construction sites to prevent accidents during heat waves. This observation is made by Xiang et al. [1] as well when they compared accidents during heat waves in several industries. A threshold temperature of 37.7 °C was estimated for construction where the changes to accident rate occurs [1]. The studies of [33,34] also observed less accidents during heat waves compared to normal work days in summer months. The reduction of accidents could be attributed to behavioural changes of workers and preventive measures implemented by the company [9]. Workers automatically adjust their work pace to reduce excessive strain [19]. Though self-regulation is an effective method to manage heat stress, purported productivity losses prevent that from happening in construction sites where sub-contracting is a norm rather than exception and payment for work is based on the progress achieved [19,35]. In addition, organizational culture plays a role in shaping the workers’ response to heat stress [20]. For example, a very high power distance and risk taking attitude prevalent in some Asian countries prevent workers from challenging the status quo of the work process and inhibit self-regulation [20]. Workers tend to “accept the work pressure at the expense of safe working procedures” ([20], p. 188). The masculine culture of the construction industry displays an underestimation of heat stress risks and overestimation of one’s ability to cope with it [21]. Therefore, preventive interventions become essential to avoid workers being exposed to excessive heat stress during heat waves. These preventive measures are mainly guided by safety regulations, code of practices, and union work agreements. 

Australian health and safety regulations and code of practice recognizes the impact of heat stress on health and safety of workers. SafeWork Australia (2011), recommends an ambient temperate of 20–26 °C to provide optimum thermal comfort for workers depending on the time of the year and clothing worn [36]. It also notes that workers involved in manual work usually prefer a lower temperature range. According to the code, thermal comfort is affected by many factors, including air temperature, air movement, humidity, clothing, the amount of physical exertion, average temperature of the surroundings, and sun penetration ([36], p. 14). As heat waves are very frequent and increasing in South Australia, construction workers through the Master Builders Association Combined Unions Compliance Agreement have resorted to a common work procedure to mitigate excessive heat stress at the onset and during heat waves. According to this agreement work areas in a site is divided into the three following categories:

1. Exposed areas such as exposed work platforms, outdoor work etc. which are directly affected by ambient heat.
2. Less exposed areas—areas cooler than the general outside temperature (e.g., lower levels of multi-storey buildings, inside or shaded areas, basements, etc.).
3. Air conditioned areas.

During the onset of a heat wave where the temperature is raising but less than 35 °C, actions need to be taken to minimize heat discomfort and stress of workers in exposed areas. In addition, these workers need to be progressively transferred to appropriate work in less exposed areas. Work in the other two areas will continue normally. When the outside temperate reaches 35 °C, work in exposed areas should cease except where needed to complete concrete pours or emergency work. Workers are entitled to double time rates during these operations. Work in less exposed areas will continue normally but workers can stop work one hour early. However, work in air conditioned areas will continue normally. When the temperature reaches 37 °C, all work in less exposed areas also should cease. The work in air conditioned areas will continue normally. In addition to these agreed work procedures, SafeWork Australia (2011) promotes some best practices to reduce heat strain and heat exhaustion, such as ([36], p. 14). These measures could be categorized broadly into the following three suggestions: improved ambient conditions using mechanical means (e.g., fans, coolers); isolation of workers from the heat source (e.g., altering work schedule, rest breaks, job rotation, slowing down); and encourage light clothing. 

This study captures some of the most vulnerable groups and work activities that need additional attention by WHS authorities and construction companies. Workers in the civil engineering sub-sector were found to be highly vulnerable to accidents in comparison to their building, building services, and general construction counterparts. Their exposure to accidents is approximately 2.4 times higher during heat waves compared to control periods. According to Lundgren et al. (2013), workers in outdoor occupations are the most vulnerable to heat waves [25]. Workers in civil construction sites do not have the luxury of shade and protection of the structure itself once it is partly completed, like the basement and lower levels of a multi-storey building [19]. Road construction workers are more vulnerable to radiant heat determined by the open site characteristics [19]. The personal protective equipment (PPE) worn by these workers also severely impedes heat exchange through evaporation [25]. Measurements undertaken in Hong Kong revealed 57 °C of air temperature inside a workers’ helmet when the environmental temperature was only 33 °C [19]. Workers are tempted to take off PPE due to severe heat stress exposing themselves for accidents and injury [19,25]. The study highlights the vulnerability of older workers to heat waves with relatively higher occurrence and severity among this cohort. The mean expenditure for injuries was more than double during the heat wave period. Xiang et al. (2013) reported similar observations for the age group of 55 and above [1]. Similarly, workers from smaller companies are more vulnerable to accidents during heat waves compared to their larger counterparts. It is likely that smaller companies may be less effective in managing WHS as well as complying with the standard heat wave work procedures discussed above. Therefore, implementation of adequate preventive measures in small-sized companies could be a priority of WHS authorities. 

While generic guidelines and work procedures are in place to mitigate heat stress in Australia, a lack of targeted interventions specifically tailored to the abovementioned vulnerable groups and work activities is a concern. One of the implications of this research is to highlight the need for such well-focused interventions in order to protect the most vulnerable. For example, as civil construction sites are often dispersed on a large geographical area unlike building sites, some of the general preventive measures might not be suitable to mitigate exposure of workers to excessive heat. Climate change and increases in extreme weather conditions will push the governments and builders alike to find innovative ways of protecting workers from accidents and severe injuries. Further research is needed to unearth the mechanisms through which heat stress can affect workers’ exposure to accidents and ways and means of reducing such exposure. The study showed no statistically significant difference between worksites in Adelaide CBD and its suburbs during heat waves, refuting the hypothesis that heat island phenomena could exacerbate heat stress in high-density urban construction sites. Built-up areas typically absorb and emit a higher volume of long wave radiation in addition to their capacity to store heat for an extended period of time. Therefore, the ambient temperature of these built-up areas is relatively higher than the suburbs and the regional farmland [25]. Given that Adelaide represents a typical case study of a dense CBD surrounded by parklands that separates the CBD from its suburbs, one could expect the number and severity of injuries during heatwaves to be significantly higher in CBD-based worksites. However, the study could not discriminate the vulnerability of workers based on worksite location. 

The findings conclude that three aspects require meticulous scrutiny and constant monitoring to reduce severe accidents on construction sites during heat waves, including: heat stress management in civil engineering sites, small-sized projects and safety implementations, and older workers and the type of work they undertake. Controlling the level of injury severity during heat waves can benefit workers by reducing their sufferings and can benefit builders by sustaining their reputation and turnover in projects. In this regard, the findings can help Safe Work South Australia, who is the owner of the accident database used in the study, in directing/redirecting its focus for accident minimization in the construction industry. The findings can also inform OHS policies and management systems of construction companies. Although, the study was conducted in the context of South Australia, the findings can be generalized and applied to the construction sector in other parts of Australia or even overseas that possess similar environmental characteristics.

While the study makes several contributions to the practice of heat stress management in construction projects, some limitations should be noted. The major limitation point is in regards to the use of a claims database to obtain injury data during heat waves and control periods [37,38]. Such data would not allow rigorous in-depth analyses compared to other tools such as case studies [24,39,40,41], questionnaire surveys [42,43,44,45], or interviews [40]. Second, all variables associated with an accident cannot be included in the analysis due to the limited number of variables reported in a database. Third, all accidents occurring during the period under consideration would not be reported, especially the minor accidents which do not benefit from claims or fatalities for which no next-of-kin are available to report. In addition, with regard to the use of claim databases, people working in the informal sector and as sole traders or partnerships would not have insurance policies, and those accidents would be totally missed from reporting [37]. Thus, underreporting can create a bias in the sample that is being analysed [38,46]. However, claims databases are much superior compared to government databases, as the latter does not have a reward or incentive inbuilt for reporting [38]. Despite these limitations, the major advantage of using claims databases is the large sample size. The present study uses 29,438 compensation claims and the large sample size provides an opportunity to use statistical tools to generalize the results.

## 5. Conclusions

The Construction Industry worldwide is being criticized for its health and safety record. Accidents and injuries in construction projects cause suffering to victims as well as loss of productivity. It is argued that heat stress increases the risk of occupational accidents and injuries. Heat stress can have a profound and progressive effect on construction workers’ health ranging from heat rash, heat cramp, heat fainting, and heat exhaustion, to heatstroke leading to morbidity and mortality in some instances. It is projected that heat wave events in South Australia will increase in the future due to climate change. Therefore, more empirical evidence is needed on how heat wave events can impact construction accidents and their severity. This paper investigated the impact of heat stress on accidents using statistical analyses. The statistical analyses were carried out on a database containing compensation claims reported in the period from 2002 to 2013 within the South Australian Construction Industry. The results showed that worker characteristics, type of work, work environment, and agency of accident governs the severity. It was found that workers in the civil engineering sub-sector are more highly vulnerable to accidents and injuries than building, building services, and general construction workers. Old age workers are more vulnerable to accidents and more likely to suffer higher severity of accidents than younger workers during heat waves. Similarly, small-sized companies are over-represented among severe construction accidents during heat wave periods. WHS policies and practice of adaptation and preventative measures should be aimed at small-sized companies and civil engineering construction workers, particularly targeting those of old age.

## Figures and Tables

**Table 1:** Occurrence of accidents during heat wave and control periods for worker characteristics.

Factor	Category	Heat Wave Period		Control Period	
		Number of Accidents	(%)	Number of Accidents	(%)
Age	Less than 25	135	19.4	135	18.8
	25–34	178	25.6	169	23.5
	35–44	167	24.0	180	25.0
	45–54	130	18.7	152	21.1
	55 and above	86	12.4	84	11.7
Experience	Experienced	552	79.1	558	77.5
	New	146	20.9	162	22.5
Gender	Male	684	98.0	695	96.5
	Female	14	2.0	25	3.5

**Table 2:** Injury severity during heat wave and control periods for worker characteristics.

Factor	Category	Heat Wave Period			Control Period		
		Minor (%)	Major (%)	Mean Cost (AUS$)	Minor (%)	Major (%)	Mean Cost (AUS$)
Age	Less than 25	20.4	16.9	6578	19.8	15.1	9483
	25–34	25.2	26.4	9371	22.0	27.9	15,408
	35–44	24.3	23.1	30,186	24.3	27.4	23,084
	45–54	18.7	18.4	11,420	22.0	18.4	15,355
	55 and above	11.4	15.2	26,381	11.9	11.2	12,747
Experience	Experienced	74.4	85.1	14,290	72.2	87.9	15,651
	New	25.6	14.9	23,806	27.8	12.1	16,733
Gender	Male	97.9	96.6	16,368	97.6	93.9	16,052
	Female	2.1	3.4	12,000	2.4	6.1	11,323

**Table 3:** Occurrence of accidents during heat wave and control periods for type of work.

Factor	Category	Heat Wave Period		Control Period	
		Number of Accidents	(%)	Number of Accidents	(%)
Sub-sector	General Construction	59	8.5	77	10.7
	Building	272	39.0	309	42.9
	Civil	109	15.6	46	6.4
	Construction Services	258	37.0	288	40.0
Occupation	Admin staff	29	4.2	32	4.4
	Bricklayer	33	4.7	29	4.0
	Carpenter	55	7.9	53	7.4
	Electrician	82	11.7	75	10.4
	Landscaper	20	2.9	24	3.3
	Mechanic	73	10.5	58	8.1
	Painter	16	2.3	21	2.9
	Plumber	43	6.2	44	6.1
	Plant operator	64	9.2	64	8.9
	Plasterer	21	3.0	27	3.8
	Roofer	10	1.4	10	1.4
	Supervisor	42	6.0	43	6.0
	Steel worker	6	0.9	12	1.7
	Unskilled worker	160	22.9	173	24.0
	Other	44	6.3	55	7.6

**Table 4:** Injury severity during heat wave and control periods for type of work.

Factor	Category	Heat Wave Period			Control Period		
		Minor (%)	Major (%)	Mean Cost (AUS$)	Minor (%)	Major (%)	Mean Cost (AUS$)
Sub-sector	General Construction	10.7	6.9	17,064	8.1	9.1	12,618
	Building	36.8	49.4	22,045	41.1	51.5	20,175
	Civil	15.2	19.5	16,646	5.6	11.1	28,496
	Construction Services	42.6	24.1	9868	39.9	28.3	10,165
Occupation	Admin staff	4.5	6.1	6828	2.8	4.7	15,553
	Bricklayer	0.6	2.0	4998	0.9	0.0	5692
	Carpenter	5.0	7.1	32,980	4.5	8.1	30,794
	Electrician	10.5	7.1	13,382	8.2	4.7	17,402
	Landscaper	3.6	3.1	24,101	1.5	3.5	14,782
	Mechanic	5.0	4.1	9865	5.1	0.0	9436
	Painter	3.2	3.1	3126	1.8	0.0	9951
	Plumber	10.1	8.2	9705	6.3	9.3	7973
	Plant operator	9.3	6.1	24,773	7.1	11.6	17,029
	Plasterer	3.9	2.0	5507	1.4	9.3	3731
	Roofer	0.7	1.0	5148	0.6	1.2	14,157
	Supervisor	4.9	3.1	21,353	2.7	4.7	11,792
	Steel worker	4.5	3.1	21,406	2.6	4.7	11,476
	Unskilled worker	24.3	36.7	17,701	50.0	29.1	23,451
	Other	9.9	7.1	22,230	4.6	9.3	26,480

**Table 5:** Occurrence of accidents during heat wave and control periods for work environment.

Factor	Category	Heat Wave Period		Control Period	
		Number of Accidents	(%)	Number of Accidents	(%)
Size of company	Small	254	36.4	249	34.6
	Medium	330	47.3	334	46.4
	Large	114	16.3	137	19.0
Worksite Location	CBD *	103	17.0	103	16.1
	Suburb	503	83.0	536	83.9
Agency of Accident	Material	175	25.1	182	25.3
	Structure	33	4.7	41	5.7
	Electricity	9	1.3	22	3.1
	Equipment	92	13.2	92	12.8
	Environment	33	4.7	30	4.2
	Small tool	91	13.0	102	14.2
	Vehicle	82	11.7	75	10.4
	Work platform	69	9.9	51	7.1
	Other	114	16.3	125	17.4

* CBD: Central Business District.

**Table 6:** Injury severity during heat wave and control periods for work environment.

Factor	Category	Heat Wave Period			Control Period		
		Minor (%)	Major (%)	Mean Cost (AUS$)	Minor (%)	Major (%)	Mean Cost (AUS$)
Size of company	Small	48.3	52.9	23,442	34.4	43.4	19,466
	Medium	48.2	35.6	14,243	46.3	38.4	15,700
	Large	18.3	11.5	6220	19.3	18.2	9877
Worksite location	CBD	4.7	4.7	28,661	7.4	5.6	20,027
	Suburb	95.3	95.3	14,608	92.6	94.4	15,552
Agency of accident	Material	24.7	22.1	12,352	27.2	18.4	12,221
	Structure	4.6	4.7	22,745	6.1	5.1	12,426
	Electricity	1.3	1.2	42,708	3.0	3.1	20,763
	Equipment	14.6	11.6	16,862	12.6	19.4	19,859
	Environment	4.4	4.7	14,091	4.8	1.0	8511
	Small tool	14.6	9.3	8149	15.7	6.1	7918
	Vehicle	12.1	14.0	13,726	10.7	11.2	11,586
	Work platform	10.4	12.8	25,208	6.1	14.3	30,078
	Other	13.1	19.8	21,439	13.7	21.4	23,683
